# T cell immune awakening in response to immunotherapy is age-dependent

**DOI:** 10.1016/j.ejca.2021.11.015

**Published:** 2022-02

**Authors:** Zena Salih, Antonia Banyard, Joshua Tweedy, Elena Galvani, Philippa Middlehurst, Sarah Mills, John Weightman, Avinash Gupta, Paul C. Lorigan, Cong Zhou, Nathalie Dhomen, Sara Valpione, Richard Marais

**Affiliations:** a Molecular Oncology Group, Cancer Research UK Manchester Institute, The University of Manchester, Alderley Park, Macclesfield, Cheshire SK10 4TG, United Kingdom; b The Christie NHS Foundation Trust, 550 Wilmslow Road, Manchester M20 4BX, United Kingdom; c Flow Cytometry, Cancer Research UK Manchester Institute, the University of Manchester, Alderley Park, Macclesfield, Cheshire SK10 4TG, United Kingdom; d Manchester Cancer Research Centre Biobank, The Christie NHS Foundation Trust, 550 Wilmslow Road, Manchester M20 4BX, United Kingdom; e Molecular Biology Core Facility, Cancer Research UK Manchester Institute, the University of Manchester, Alderley Park, Macclesfield, Cheshire SK10 4TG, United Kingdom; f Division of Cancer Sciences, The University of Manchester, Oxford Road, Manchester M13 9PL, United Kingdom; g Cancer Biomarker Centre, Cancer Research UK Manchester Institute, The University of Manchester, Alderley Park, Macclesfield, Cheshire SK10 4TG, United Kingdom

**Keywords:** Immunotherapy, Immune-checkpoint blockade, Age, Melanoma, T cell receptor, T cell

## Abstract

**Background:**

Precision immuno-oncology approaches are needed to improve cancer care. We recently demonstrated that in patients with metastatic melanoma, an increase of clonality or diversity of the T cell receptor (TCR) repertoire of peripheral T cells following one cycle of immunotherapy is coincident with response to immune-checkpoint blockade (ICB). We also identified a subset of peripheral CD8^+^ immune-effector memory T cells (T_IE_ cells) whose expansion was associated with response to ICB and increased overall survival. To improve our understanding of peripheral T cell dynamics, we examined the clinical correlates associated with these immune signatures.

**Methods:**

Fifty patients with metastatic melanoma treated with first-line anti-PD-1 ICB were included. We analysed TCR repertoire and peripheral T_IE_ cell dynamics by age before treatment (T0) and after the first cycle of treatment at week 3 (W3).

**Results:**

We observed a correlation between T_IE_ abundance and age at T0 (r = 0.40), which reduced following treatment at W3 (r = 0.07). However, at W3, we observed two significantly opposing patterns (p = 0.03) of TCR repertoire rearrangement in patients who responded to treatment, with patients ≥70 years of age showing an increase in TCR clonality and patients <70 years of age showing an increase in TCR diversity.

**Conclusions:**

We demonstrate that immunotherapy-induced immune-awakening patterns in patients with melanoma are age-related and may impact patient response to ICB, and thus have implications for biomarker development and planning of personalised therapeutic strategies.

## Highlights

• Immune-effector T cell expansion early during immunotherapy predicts tumour shrinkage.
• Immune-checkpoint blockade drives age-dependent bifurcated evolution of T cell clonality or diversity.
• T cell evolution provides a convenient early biomarker of response to immunotherapy.

## 1 Introduction

To facilitate the identification of new therapeutic strategies and personalised treatments for patients with melanoma, we need to understand the biological mechanisms that underpin why some patients benefit from immune-checkpoint blockade (ICB) therapies, whereas others do not. Ideally, therapy decisions are based on patient-specific cancer or immunological biomarkers, but many factors are known to influence how patients respond to ICB (stage, lactate dehydrogenase [LDH] level, organ system involved). Furthermore, age-related thymic atrophy reduces naïve T cell output, which could affect T cell diversity and cause age-related immunodepression, leading to different patterns of response to ICB [[1], [2], [3], [4], [5]]. Thus, to deliver effective precision immune-oncology, a variety of clinical variables need to be considered for clinical decision making.

We recently analysed peripheral blood from patients with melanoma before treatment and after the first cycle of ICB [6]. We reported that patients who went on to respond to ICB therapy presented an increase either in the clonality or diversity of the T cell receptors (TCRs) on their circulating T cells. Patients who failed to respond to treatment did not develop this dichotomised response in their TCR. We also reported that after their first cycle of treatment, patients who responded to ICB presented an expansion of a subset of peripheral CD8^+^ memory T cells that were characterised as CD27^−^/CCR7^−^, bore the characteristics of cytotoxic T cells and are known to infiltrate tumours. Because this T cell subset was associated with response to ICB therapy, we called them T immune effector or T_IE_ cells.

Here, we examined the clinical characteristics in patients with melanoma treated with first-line anti-PD-1 ICB. We observed age-related effects on TCR repertoire evolution and T_IE_ cell expansion that are consistent with age-related changes that occur in the human immune system. Our data show that age influences T cell awakening by ICB therapy and, therefore, that age must be incorporated into the development of biomarkers to monitor responses to immunotherapy.

## 2 Patients and methods

**Patient samples**. Blood samples from patients and healthy donors were collected under the Manchester Cancer Research Centre (MCRC) Biobank ethics application #07/H1003/161+5, ethics code 18/NW/0092, with written informed consent from the patients at The Christie NHS Foundation Trust. The study was approved by MCRC Biobank Access Committee application 13_RIMA_01. All clinical investigations were conducted according to the principles expressed in the Declaration of Helsinki and good clinical practice guidelines. In the present study, we re-analysed the data from our previously reported cohort [6], which included a total of 50 patients with metastatic melanoma, treated with either single-agent pembrolizumab or combination nivolumab plus ipilimumab as first-line therapy. Inclusion criteria included treatment naïve, inoperable locally advanced or metastatic melanoma. Patients were excluded if they had received any systemic oncological treatment in the neoadjuvant, adjuvant or metastatic setting for melanoma or other cancers, concomitant therapy with immunosuppressant drugs at enrolment or had synchronous other active malignancies. As a surrogate measure of tumour burden, the sum of target lesions on baseline and week 12 (W12) radiographic CT scans were calculated using RECIST 1.1. Measurements were available for 36 patients; the different number of patients included in the sub-studies reflects the availability of detailed target metastatic lesion measurements in the scan reports.

### 2.1 Peripheral T cell, T cell receptor and cell-free DNA analysis

Data from our previously reported cohort [6] were analysed. Sample collection and processing were performed as previously described [6]. TCR sequence data were retrieved for 29 (peripheral blood mononuclear cell, PBMC) and 28 (cell-free DNA, cfDNA) of the 50 patients.

### 2.2 Statistical analyses

Correlation between continuous variables was performed with the Spearman test; the Spearman r was reported as a measure of the correlation magnitude. Linear discriminant analysis was used to separate the Δ_W3_ Renyi index and Δ_W3_ Gini coefficient and categorise the values in classes as indicated in the Fig. 3 legend. Mann–Whitney U test (two-sided) was used for comparison between continuous variables. Comparison between categorical variables was performed with Fisher's exact test. All tests were two-sided, and p-values <0.05 were considered significant. Analyses were performed using Prism version 7.0.

## 3 Results

### 3.1 Patient cohort

We recruited 50 predominantly male (32 male, 18 female) patients with treatment-naïve metastatic melanoma attending The Christie Hospital NHS Foundation Trust for first-line immunotherapy [6]. Just over half of the patients (54%) had stage M1c disease, 16% of patients (8/50) had baseline LDH > ULN (upper limit normal), the median age was 70 years (range: 35–85), and 68% of patients (34/50) were *BRAF*-wild type (Table 1). The number of metastatic sites ranged from 1 to 7, and of the 27 patients with stage M1c or M1d disease (Table 1), 15 patients had hepatic metastases, 2 patients in combination with cerebral metastases. Patients received first-line single-agent anti-PD1 drugs (200 mg pembrolizumab 3 weekly, or 480 mg nivolumab 4 weekly, 29 patients) or combination anti-PD1 plus anti-CTLA4 (1 mg/kg nivolumab plus 3 mg/kg ipilimumab 3 weekly for 4 doses, followed by 3 mg/kg nivolumab 2 weekly; 21 patients) as per standard of care. Assessment of tumour response was performed by computed tomography (CT) scans at week 12 (W12).

**Table 1:** **Clinical characteristics of the patient cohort**. The table summarises the clinical characteristics of the patient cohort. LDH = lactate dehydrogenase, RECIST = radiologic evaluation criteria for solid tumours; W12 RECIST 1.1 = CT scan lesion measurements of metastatic sites at week 12 from treatment start. ∗ The different number of patients included in the sub-study reflects the availability of detailed target metastatic lesion measurements in the scan reports.

Clinical variable	Number (%)	Median (range)	Total number of patients evaluated
Gender			50
Male	32 (64%)		
Female	18 (36%)		
Stage			50
IIIC – M1a	10 (20%)		
M1b	13 (26%)		
M1c – M1d	27 (54%)		
BRAF V600E/K			50
Mutated	16 (32%)		
Wild type	34 (68%)		
Age (years)		70 (35–85)	50
Baseline LDH (IU/L)		371 (165–2987)	50
<ULN	42 (84%)		
>ULN	8 (16%)		
Treatment			50
Single agent αPD1	29 (58%)		
Combination αPD1 + αCTLA4	21 (42%)		
Sum of RECIST 1.1 marker lesion diameters at Baseline (cm)		4.9 (1.1–21.5)	37∗
Sum of RECIST 1.1 marker lesion diameters at W12 (cm)		4.5 (0–31.1)	36∗
Number of organ sites with metastases		2 (1–7)	39∗

### 3.2 Patient response to ICB correlates with an expansion in peripheral T_IE_ cells

**Fig. 1:**
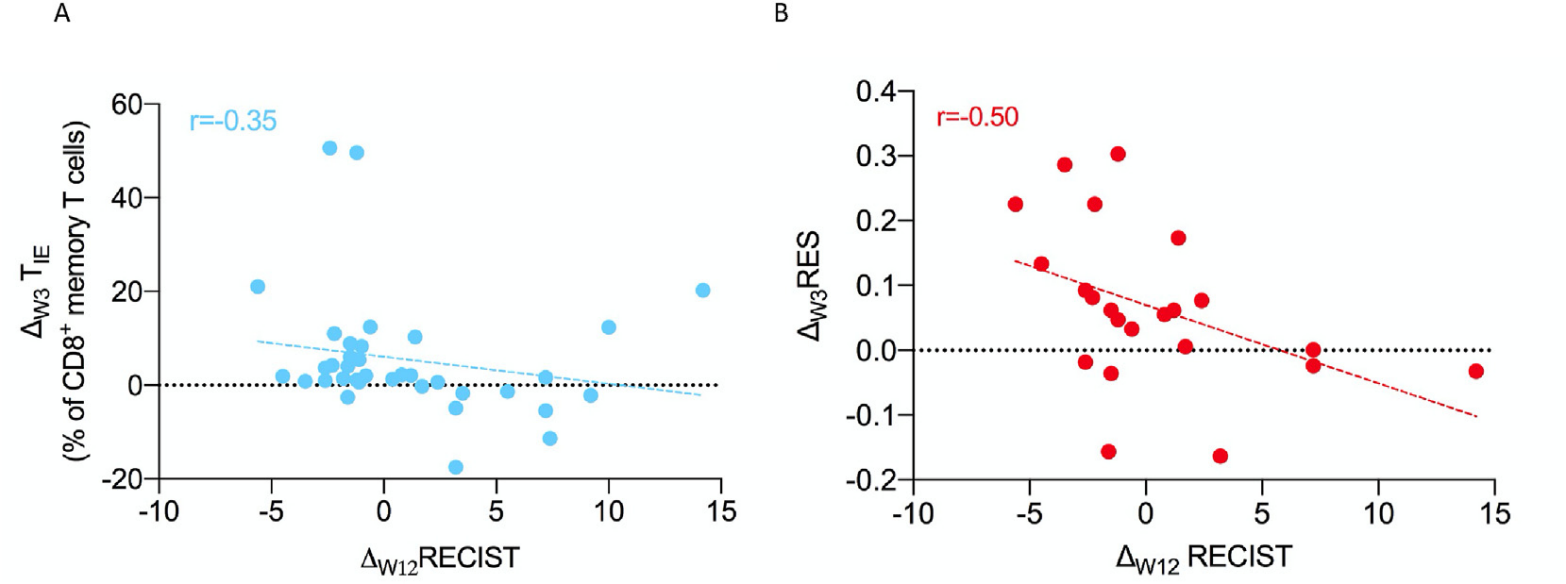
**Correlation between radiological response and peripheral biomarkers**. **A**: Correlation (r = −0.35) between Δ_W12_RECIST and Δ_W3_T_IE_ in the melanoma patient cohort (n = 36). **B**: Correlation (r = −0.50) between Δ_W3_RES and Δ_W12_RECIST in the melanoma patient cohort (n = 22 patients with both measurements available). Data points represent individual patients. Dotted line: linear regression line.

First, we used flow cytometry to quantify the percentage of T_IE_ cells in the patients' circulating cytotoxic memory T cells from PBMC [6] before treatment (T0) and after one cycle of ICB at week 3 (W3). From this, we calculated the change in T_IE_ abundance at W3 compared with T0 (W3[T_IE_]–T0[T_IE_] = Δ_W3_T_IE_). As a surrogate of tumour burden, we calculated the sum of the measured target RECIST lesions from the patients' scans at T0 (T0[RECIST]) and week 12 (W12[RECIST]) and then calculated the change at W12 compared with T0 (W12[RECIST]–T0[RECIST] = Δ_W12_ RECIST) [7, 8]. Notably, patients with a Δ_W12_RECIST of ≤0 (tumour shrinkage) had a mean Δ_W3_T_IE_ of 9.57% (range: −2.55–50.62%), whereas patients with a Δ_W12_RECIST of >0 (tumour growth) had a mean Δ_W3_T_IE_ of 0.4% (range: −17.5–20.2%) (Fig. 1A), indicating a negative correlation between T_IE_ cell subset expansion and tumour burden changes (r = −0.35).

To evaluate T cell turnover (death), we determined the TCR rearrangement efficiency score (RES) in cfDNA from the patients' blood. The RES measures the proportion of functional TCR sequences as a product of all TCR sequences, and we recently demonstrated that the change in cfDNA TCR RES at W3 compared with T0 (W3[RES]–T0[RES] = Δ_W3_ RES) is a surrogate for T cell turnover [6]. We show here that patients with an average Δ_W3_RES of 0.02 (−0.16–0.17) had a Δ_W12_RECIST > 0, whereas patients with an average Δ_W3_RES of 0.1 (−0.15–0.30) had a Δ_W12_ RECIST ≤ 0 (Fig. 1B), indicating a negative correlation between peripheral T cell turnover and changes in tumour size (r = −0.50).

Thus, we extend our previous observations by showing that the magnitude of the peripheral T_IE_ cell expansion and the magnitude of T cell turnover at W3 are both inversely proportional to the change in tumour burden at W12 (r = −0.35, r = −0.50, respectively) (Fig. 1A and B).

### 3.3 T cell kinetics in response to ICB are not affected by age

We next investigated how other clinical factors affected T_IE_ cell expansion and T cell turnover by comparing T_IE_ cell abundance and TCR RES at T0 and W3 across established clinical parameters. We did not find significant association between T_IE_ cell abundance at T0 or W3, or TCR RES at T0 or W3, and gender (p = 0.76, p = 0.93, p = 0.49, p = 0.75), American Joint Committee on Cancer [AJCC] 8^th^ edition stage of disease (p = 0.07, p = 0.09, p = 0.81, p = 0.80), BRAF V600E/K mutation status (p = 0.68, p = 0.1, p = 0.92, p = 0.19), or LDH (p = 0.65, p = 0.27, p = 0.29, p = 0.91) (Table 2). We were, however, intrigued to find an apparent association between treatment group and both T_IE_ cell abundance at T0 (p = 0.02) and TCR RES at W3 (p = 0.01) (Table 2) because connected to this was a significant selection bias of preferential allocation of combined therapy to younger (mean age 58 years; range: 35–79) and single-agent therapy to older patients (mean age 73 years; range: 51–85) (p < 0.0001; Supplementary Fig. 1).

**Table 2:** **Patient cohort clinical variables and their correlation with peripheral T**_**IE**_**cells and RES at T0 and W3**. The table summarises the values of T immune effector (T_IE_) cell percental abundance in the peripheral CD8^+^ memory T cells and rearrangement efficiency score (RES) in cell-free DNA before treatment (T0) and after 3 weeks on treatment (W3) across the clinical factors. αPD1 = anti-PD1 therapy (pembrolizumab or nivolumab); αCTLA4 = anti-CTLA4 therapy (ipilimumab)**;** ULN = upper limit normal; n = number of patients (total number = 50 for the T_IE_ analyses and 28 for RES analyses due to sample availability); p is Mann–Whitney U test two-sided p or non-parametric Analysis of Variance, values in brackets indicate the variable value range.

	Gender			BRAF V600E/K			Stage				Baseline LDH			Treatment		
	Female n = 18	Male n = 32	p value	Mutant n = 16	wild type n = 34	p value	IIIC/M1a n = 10	M1b n = 13	M1c/d n = 27	p value	<ULN N = 42	>ULN n = 8	p value	αPD1 n = 21	αPD1 + αCTLA4 n = 29	p value
**T0 T**_**IE**_ mean (range)	15.19 (1.2–41.7)	17.53 (1.48–75.6)	0.76	16.37 (2.38–39.8)	16.84 (1.2–75.6)	0.68	9.06 (1.2–33.1)	24.58 (2.38–75.6)	15.72 (2.54–44)	0.07	16.94 (1.2–75.6)	15.36 (2.54–44)	0.65	20.83 (2.54–75.6)	10.97 (1.2–38.1)	0.02
**W3 T**_**IE**_ mean (range)	21.83 (3.58–79.2)	23.06 (1.2–73.4)	0.93	27.71 (8.97–69.3)	20.22 (1.2–79.2)	0.10	13.75 (4.52–30.1)	33.61 (4.37–79.2)	20.60 (1.2–69.3)	0.09	22.55 (3.58–79.2)	19.68 (0.23–69.3)	0.27	23.30 (1.2–73.4)	21.67 (4.37–79.2)	0.30

	**Female n = 11**	**Male n = 17**	**P value**	**Mutant n = 7**	**Wild type n = 21**	**p value**	**IIIC/M1a n = 5**	**M1b n = 7**	**M1c/d n = 16**	**p value**	**<ULN n** = **23**	**>ULN n = 5**	**p value**	**αPD1 n = 19**	**αPD1 + αCTLA4 n = 9**	**p value**
**T0 RES** mean (range)	0.61 (0.47–0.71)	0.64 (0.38–0.84)	0.49	0.63 (0.47–0.77)	0.62 (0.38–0.84)	0.92	0.64 (0.51–0.71)	0.65 (0.53–0.78)	0.62 (0.38–0.85)	0.81	0.64 (0.39–0.84)	0.57 (0.48–0.71)	0.29	0.61 (0.38–0.84)	0.65 (0.47–0.84)	0.53
**W3 RES** mean (range)	0.70 (0.51–0.80)	0.68 (0.52–0.85)	0.75	0.73 (0.58–0.80)	0.67 (0.51–0.84)	0.19	0.68 (0.53–0.77)	0.67 (0.51–0.78)	0.69 (0.52–0.84)	0.80	0.69 (0.51–0.85)	0.66 (0.55–0.80)	0.91	0.65 (0.51–0.85)	0.75 (0.61–0.82)	0.01

**Fig. 2:**
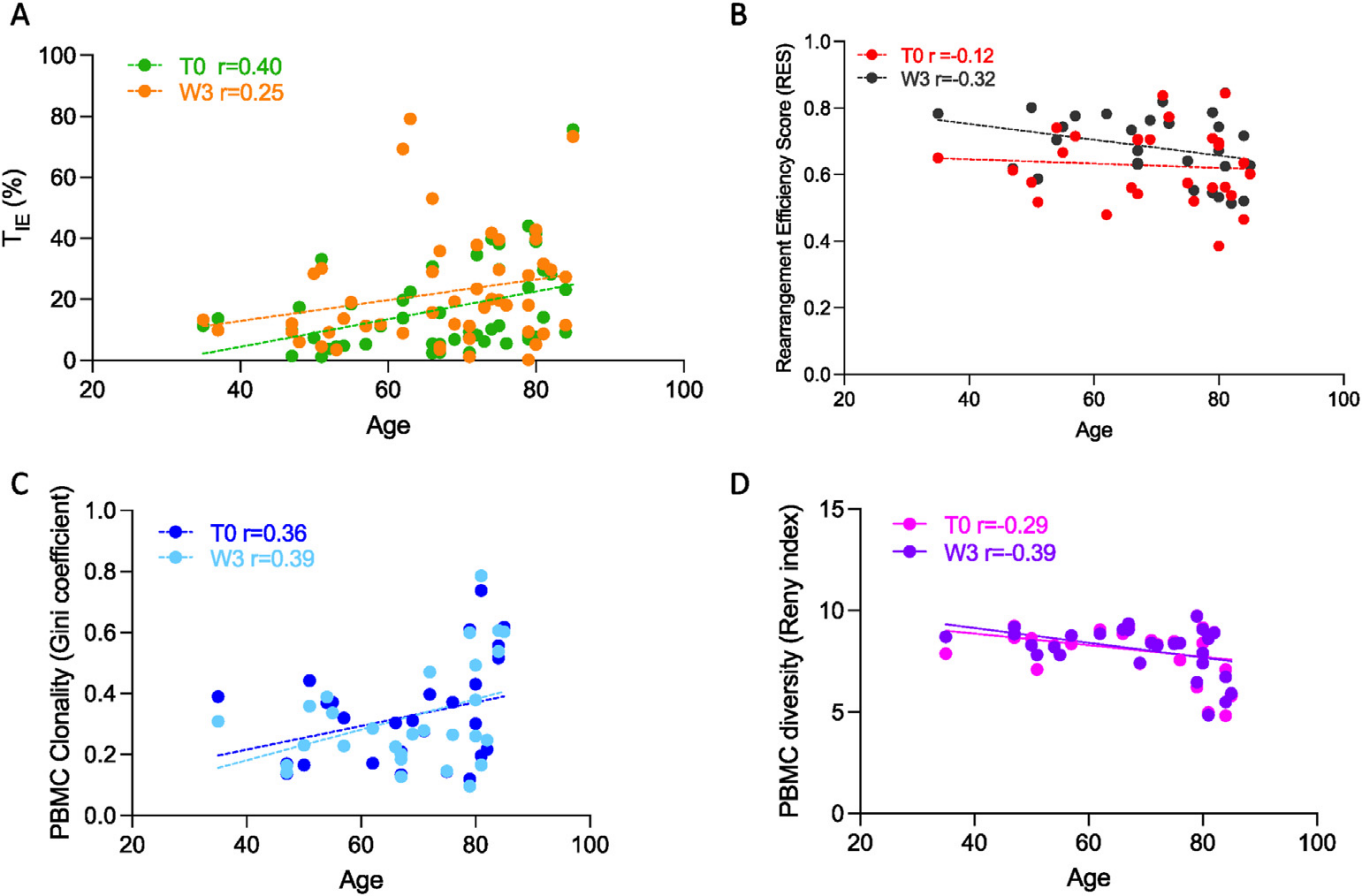
**Correlation between age and peripheral T cell biomarkers**. **A**: Correlation between age (years) and T_IE_ cell abundance at baseline (T0, green, r = 0.40; n = 50) and after first cycle of immunotherapy (W3, amber, r = 0.25; n = 50). **B**: Correlation between age (years) and TCR receptor rearrangement efficiency score (RES) at baseline (T0, red, r = −0.12; n = 28) and after the first cycle of immunotherapy (W3, black, r = −0.32; n = 28). **C**: Correlation between age (years) and PBMC clonality (Gini coefficient) at baseline (T0, navy, r = 0.36; n = 29), and after the first cycle of immunotherapy (W3, light blue, r = 0.39; n = 29). **D**: Correlation between age (years) and PBMC T cell receptor diversity (Renyi index) at baseline (T0, pink, r = −0.29; n = 29) and after first cycle of immunotherapy (W3, purple, r = −0.39; n = 29). T_IE_, immune-effector T cells; PBMC, peripheral blood mononuclear cell.

Using the accepted geriatric oncology definition for the elderly population of 70 years of age [9], we show that at T0, the mean T_IE_ cell abundance was 11.2% (range: 1.2%–33.1%) of the circulating CD8+ memory in patients of 69 years and less but 22% (range: 2.54%–75.6%) in patients who were 70 years and older (Fig. 2A). Thus, as a proportion of the memory T cell pool, the T_IE_ cell subset increased with age (r = 0.4), consistent with our observed association between treatment group and T_IE_ cells abundance at T0 (Table 2). At W3, we observed a similar pattern, with a mean T_IE_ cell abundance of 21% (range: 3.47%–79.2%) of the circulating CD8+ memory T cells in patients of 69 years and less and 24% (range: 0.23%–73.4%) in patients of 70 years and older (Fig. 2A). Consistent with an increase in T_IE_ cell abundance associated with clinical response to immunotherapy [6], across all ages, we observe an upward shift of the regression line at W3 compared with T0 (Fig. 2A).

We also show that at T0, the mean RES was 0.62 (range: 0.47–0.74) in patients of 69 years and less, and 0.62 (range: 0.38–0.84) in patients of 70 years and older, while at W3, the mean RES was 0.71 (range: 0.58–0.80) in patients of 69 years and less, but 0.65 (range: 0.51–0.84) in patients of 70 years and older (Fig. 2B). Again, consistent with an increase in RES in responding patients [6] at all ages, we observe an upward shift of the line of regression at W3 compared with T0. Although the inverse relationship between RES and age appears to be rather weak (r = −0.12 to −0.32), the slight increase in correlation between RES and age after treatment is consistent with the observed association between treatment protocol and TCR RES at W3 (Table 2).

### 3.4 TCR repertoire evolution in response to ICB is affected by age

We next examined if peripheral T cell TCR repertoire rearrangements in response to ICB therapy, another feature of immune awakening [6], was affected by established patient and tumour factors. We reconstructed PBMC TCR sequences using ImmunoSeq and applied our algorithm [6] to calculate clonality (Gini coefficient) and diversity (Renyi index). We did not find an association between Gini coefficient, at T0 and W3, or Renyi index, at T0 and W3 and gender (p = 0.21, p = 0.38, p = 0.27, p = 0.38), AJCC stage of disease (p = 0.63, p = 0.78, p = 0.99, p = 0.72), BRAF V600E/K mutation status (p = 0.82, p = 0.57, p = 0.90, p = 0.94) or LDH levels (p = 0.52, p = 0.89, p = 0.76, p = 0.72) (Table 3).

**Table 3:** **Patient cohort clinical variables and their correlation with peripheral TCR repertoire at T0 and W3**. The table summarises the value of peripheral T cell clonality (Gini coefficient) and diversity (Renyi index) before treatment (T0) and after 3 weeks (W3) on treatment across the clinical factors. αPD1 = anti-PD1 therapy (pembrolizumab or nivolumab); αCTLA4 = anti-CTLA4 therapy (ipilimumab); ULN = upper limit normal; n = number of patients; p is Mann–Whitney U test two-sided p or non-parametric analysis of variance; values in brackets are the variable range.

	Gender			BRAF V600E/K			Stage				Baseline LDH			Treatment		
	Female n = 10	Male n = 19	p value	Mutant n = 7	wild type n = 22	p value	III/M1a n = 6	M1b n = 7	M1c/d n = 16	p value	<ULN n = 24	>ULN n = 5	p value	αPD1 n = 19	αPD1 + αCTLA4 n = 10	p value
**T0 clonality** mean (range)	0.37 (0.13–0.73)	0.30 (0.11–0.61)	0.21	0.32 (0.16–0.55)	0.32 (0.11–0.73)	0.82	0.30 (0.17–0.44)	0.35 (0.19–0.61)	0.32 (0.11–0.73)	0.63	0.33 (0.12–0.74)	0.29 (0.14–0.61)	0.52	0.37 (0.13–0.73)	0.23 (0.11–0.38)	0.03
**W3 clonality** mean (range)	0.35 (0.12–0.78)	0.30 (0.09–0.60)	0.38	0.33 (0.20–0.60)	0.31 (0.09–0.78)	0.57	0.25 (0.16–0.35)	0.35 (0.16–0.60)	0.33 (0.09–0.78)	0.78	0.33 (0.10–0.79)	0.31 (0.15–0.60)	0.89	0.37 (0.12–0.78)	0.22 (0.09–0.33)	0.05
**T0 diversity** mean (range)	7.82 (4.98–9.16)	8.17 (4.82–9.73)	0.27	7.93 (4.82–9.25)	8.09 (4.98–9.73)	0.90	8.25 (7.09–9.15)	8.12 (5.78–9.29	7.95 (4.82–9.73)	0.99	8.07 (4.83–9.74)	7.99 (6.23–9.06)	0.76	7.70 (4.82–9.25)	8.72 (7.81–9.73)	0.05
**W3 diversity** mean (range)	7.92 (4.84–9.06)	8.20 (5.48–9.72)	0.38	8.11 (5.48–9.31)	8.11 (5.48–9.31)	0.94	8.49 (7.42–9.06)	8.17 (5.92–9.35)	7.93 (4.84–9.72)	0.72	8.12 (4.84–9.72)	8.08 (6.47–8.86)	0.72	7.75 (4.84–9.31)	8.79 (7.80–9.72)	0.03

We did however observe an association between treatment protocol and Gini coefficient at T0 (p = 0.03) and W3 (p = 0.05) and between treatment protocol and Renyi index at T0 (p = 0.05) and W3 (p = 0.03) (Table 3). Noting the age-dependent selection bias for treatment protocol, we examined if age impacted clonality and diversity. We show that at T0, the Gini coefficient mean was 0.26 (range: 0.13–0.44) for patients 69 years and less and 0.38 (range: 0.11–0.73) for patients of 70 years and older (Fig. 2C). At W3, the mean Gini coefficients were 0.24 (range: 0.14–0.38) for patients 69 years and less and 0.39 (range: 0.09–0.78) for patients of 70 years and older (Fig. 2C). Thus, TCR clonality showed an overall positive correlation with age (r = 0.36 and r = 0.39 at T0 and W3, respectively), but unlike T_IE_ abundance and TCR RES, the linear regression line did not shift up or down with ICB treatment but changed in slope, suggesting a trend towards increased clonality in older patients on ICB therapy (Fig. 2C).

At T0, the mean Renyi index was 8.49 (range: 7.09–9.29) for patients of 69 years and less and 7.64 (range: 4.82–9.73) in patients of 70 years and older (Fig. 2D). At W3, the mean Renyi index was 8.62 (range: 7.42–9.35) in patients 69 years and less and 7.63 (range: 4.84–9.72) in patients of 70 years and older (Fig. 2D). Thus, TCR diversity showed an inverse relationship with age (r = −0.29 and r = −0.39 at T0 and W3, respectively) and, as was observed with clonality, the linear regression line did not shift up or down with ICB treatment but changed in slope, suggesting a trend towards increased diversity in younger patients on ICB treatment (Fig. 2D).

Since our data show that peripheral T_IE_ cell expansion and TCR repertoire rearrangements were both influenced by age, we compared these variables to each other. At T0, the mean T_IE_ cell abundance was 17%, so using this as a cut-off, we show that patients with a T_IE_ cell abundance ≤17% had a mean TCR Gini coefficient of 0.29 (range: 0.11–0.73), whereas patients with a T_IE_ cell abundance >17% had a mean TCR Gini coefficient of 0.37 (range: 0.14–0.61) (Supplementary Fig. 2A). Conversely, patients with a T_IE_ cell abundance ≤17% had a mean Renyi index of 8.35 (range: 4.97–9.25), whereas patients with a T_IE_ cell abundance >17% had a mean Renyi index of 7.63 (range: 4.82–9.05) (Supplementary Fig. 2A). After one cycle of ICB treatment (W3), the mean T_IE_ cell abundance was 22%, so using this as a cut-off, we show that patients with a T_IE_ cell abundance ≤22% had a mean TCR Gini coefficient of 0.30 (range: 0.09–0.78), whereas patients with a T_IE_ cell abundance >22% had a mean TCR Gini coefficient of 0.34 (range: 0.14–0.60) (Supplementary Fig. 2B). Conversely, patients with a T_IE_ cell abundance ≤22% had a mean Renyi index of 8.23 (range: 4.84–9.72), whereas patients with a T_IE_ cell abundance >22% had a mean Renyi index of 7.93 (range: 5.48–9.31) (Supplementary Fig. 2B). Thus, both before and after one cycle of ICB treatment, there was a positive correlation between T_IE_ cell abundance and peripheral T cell TCR clonality (r = 0.43, r = 0.19, respectively), but an inverse correlation between T_IE_ cell abundance and peripheral T cell TCR diversity (r = −0.42, r = −0.17, respectively) (Supplementary Fig. 2A, B). Note however, that both relationships became weaker after one cycle of therapy because the T_IE_ cell population expanded in an inverse proportion to tumour burden changes (Fig. 1A).

**Fig. 3:**
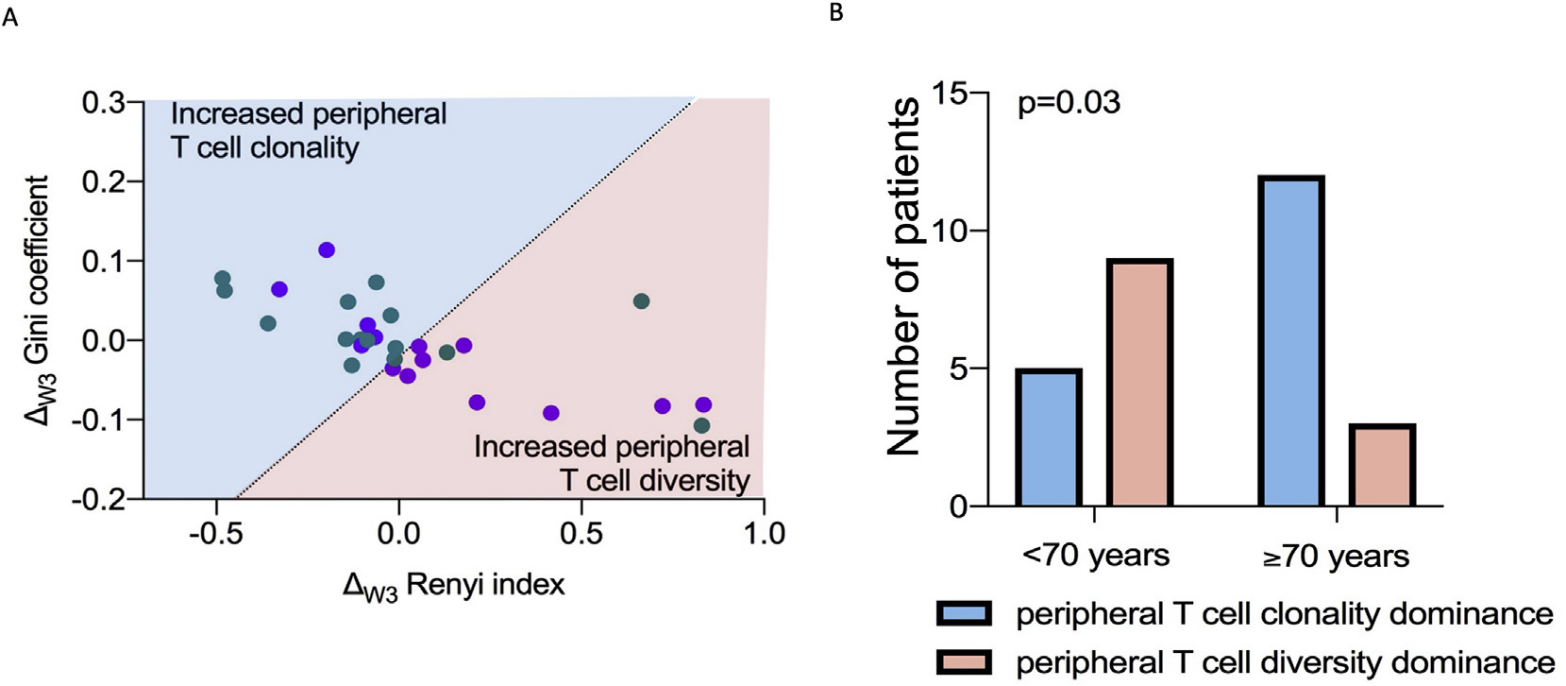
**Relationship between age and peripheral T cell TCR repertoire re-arrangement**. **A**: Scatter plot showing changes in peripheral TCR clonality (Gini coefficient) and diversity (Renyi index) after one cycle of anti-PD1-based treatment (W3 compared T0: Δ_W3_) for patients in the age group <70 years (purple dots) and ≥70 years (green dots). The dotted line represents the linear discriminant (X0 = 0.024, slope = 0.4) for TCR re-arrangement with increased peripheral T cell clonality (hemiplot in blue) versus increased peripheral T cell diversity (hemiplot in pink). Each dot is a single patient (n = 29). **B**: Comparison of the number of patients with peripheral T cell re-arrangement pattern towards dominant clonality (blue) or dominant diversity in (pink) from A, according to age group (n = 29; Fisher test p = 0.03). TCR, T cell receptor.

We previously showed that treatment induces a bifurcated outcome in peripheral T cell TCR repertoire [6], so we compared how peripheral T cell clonality and diversity changed at W3 relative to T0 (W3–T0[Gini] = Δ_W3_ Gini; W3–T0[Renyi] = Δ_W3_ Renyi). To separate the T cell TCR repertoire rearrangement at W3 according to evolution pattern (prevalence of increased clonality versus prevalence of increased diversity), we applied a linear classifier algorithm to segregate the changes of clonality and diversity at W3 into predominant clonal evolution (blue hemi-plot in Fig. 3A) or predominant diverse evolution (pink hemi-plot in Fig. 3A) for patients <70 years (purple dots in Fig. 3A) and ≥70 years (green dots in Fig. 3A). There are 5 patients <70 years and 12 patients ≥70 years who fell into the predominant clonal evolution plot, whereas 9 patients <70 years and 3 patients ≥70 years fell into the predominant diverse evolution plot (Fig. 3A). Thus, in patients <70 years of age, 5/14 had peripheral T cell TCR clonality dominance and 9/14 had peripheral T cell TCR diversity dominance (Fig. 3B), whereas in patients ≥70 years of age, 12/15 had peripheral T cell clonality dominance and 3/15 had peripheral T cell diversity dominance (p = 0.03, Fig. 3B). Thus, in response to ICB therapy, TCR rearrangements trend towards increased diversity in younger patients but increased clonality in older patients.

## 4 Discussion and conclusions

The role of age as a prognostic factor for melanoma is well described [10], but it is unclear if this is a consequence of distinct melanoma biology [11], different patterns of UV-induced DNA damage [12] or immune-senescence [13]. Notably, elderly patients often display greater benefit from ICB than younger patients [14, 15], and although the mechanisms underlying this observation are unclear [14], this could be due to selection bias caused by fitter patients with less advanced disease within the elderly cohort being offered ICB preferentially [15]. Taken together, these observations suggest that age plays an important role in the interactions between melanoma, the immune system and immunotherapy and are consistent with our findings here that age affects immune-awakening in response to ICB.

We previously reported that changes in T_IE_ abundance and the RES after the first cycle of immunotherapy identify which patients will achieve disease control at W12 [6]. Here, we extend those findings by showing that in a cohort of 50 melanoma patients receiving ICB, changes in T_IE_ cell abundance inversely correlated with changes in tumour burden determined by RECIST target lesion size in patients' CT scans. This suggests that an increase in T_IE_ abundance 3 weeks after the start of ICB therapy predicts tumour shrinkage at the W12 assessment. Similarly, we show that peripheral T cell pools undergo dynamic turnover proportional to the magnitude of response confirming that the immune signature that we have previously described [6] is a reliable early biomarker of response to ICB.

We next demonstrate that although T_IE_ expansion and peripheral T cell turnover are biomarkers of immunotherapy response across all age groups, patients in different age groups present different patterns of peripheral T cell TCR repertoire evolution in response to ICB. Specifically, after one cycle of immunotherapy, in patients ≥70 years immunotherapy tends to a preferential increase in peripheral T cell TCR clonality, whereas in patients <70 years, it tends to a preferential increase in peripheral T cell TCR diversity. In addition, we show that T_IE_ cell abundance inversely correlates with peripheral TCR repertoire diversity and directly correlates with peripheral T cell repertoire clonality, consistent with repertoire convergence in patients with pre-existing T_IE_ expansion [6]. Moreover, this relationship became less apparent as T_IE_ expansion was boosted in patients benefitting from treatment, as we previously showed [6], irrespective of age.

Our data are consistent with the observation that age is associated with decreased thymic output [[6], [7], [8], [9], [10], [11], [12], [13], [14], [15], [16]]. Age-related regression of the thymus is accompanied by a decline in naïve T cell output, which is thought to contribute to the reduced T cell diversity in older individuals and is linked to increased susceptibility to infection, autoimmune disease and cancer [2]. Although widely accepted, this age-associated TCR repertoire constriction has not been widely studied using direct methodologies [1] and has not been analysed in patients with cancer treated on immunotherapy. While it is acknowledged that high TCR repertoire diversity is a prerequisite for an effective adaptive immune response against new antigens [17] and that age impacts cancer therapy responses [18], this is the first report of age-specific differential immune-awakening patterns induced by immunotherapy in patients with cancer. We posit that our findings reflect age-related thymic involution [19, 20] and a consequent reduction of new clonotype output [21] available to recognise and kill cancer cells [22].

We observed a significant difference in age between the two treatment groups, which likely reflects selection bias in the real-world clinical setting, as we observed that patients ≥70 years were preferentially assigned to single-agent therapy (Supplementary Fig. 1). This could affect the interpretation of the effect of treatment regimen on the biomarker dynamics, and the modest size of our cohort precludes the prospect of meaningful insight from any intra-group comparisons. However, the absence of any change from pre-treatment in the relationships between treatment regimen and the immune-biomarkers we measured after treatment initiation, suggests a negligible effect of drug schedule on the T cell biomarkers we studied in our cohort. Moreover, we saw no effect of the clinical variables of gender, stage, BRAF mutation and LDH status on T_IE_ cell expansion, T cell turnover or peripheral T cell TCR repertoire rearrangements. This supports the importance of T_IE_ cell expansion, T cell turnover and peripheral T cell TCR repertoire rearrangements as biomarkers of response to therapy. Note that the LDH assay was modified during the course of this study and a change in the upper limit of normal (ULN) cut off values affected 6 of our 50 patients, which could have influenced the relationship between LDH ULN and the T cell biomarkers we report. Although limited by a small sample size and the biological biases of an unselected population, together our results support a model whereby age does not affect peripheral T_IE_ subset expansion in response to ICB but does influence immunotherapy-induced peripheral TCR repertoire evolution.

Our findings need validation in larger, randomised cohorts that can differentiate responses in younger versus older patients, controlling for the other clinical variables, but they highlight the importance of considering age during the development of immunotherapy approaches and biomarker-led strategies. For example, TCR-based biomarkers need to consider how age affects TCR repertoire evolution following treatment and therapies that require more diverse T cell repertoires may be less effective in older patients. Critically, the inconsistent recruitment of older patients into clinical trials has led to the development of treatments largely in younger patients who typically have different biological and physiological responses [23, 24]. As our data highlight, future work should focus on ensuring the inclusion of patients ≥70 years of age in immunotherapy clinical trials and the reporting of age-group specific survival outcomes. Refinements in the design of preclinical and clinical trials are necessary to determine how ageing impacts the efficacy of different classes of immunotherapy. Finally, the hypothesis deriving from our results, that new clonotype thymic output reduces with age, potentially provides a biological explanation of the bifurcated reorganisation of the TCR repertoire we have previously observed in response to immunotherapy [6].

Although our sample size is relatively small and our observations require further validation and qualification before these biomarkers could be introduced into clinical practice, our exploratory findings have potentially relevant implications for biomarker development and therapy planning. In particular, although T_IE_ cells may act as early prognostic biomarkers independent of patient age, TCR repertoire analysis must be contextualised by patient age. Moreover, therapeutic strategies that aim to boost peripheral T cell repertoire diversification to increase tumour neoantigen recognition are likely to be less effective in patients ≥70 years because successful new clonotype recruitment would be ineffective due to thymic involution.

## Author contribution

Conceptualisation: SV and ZS. Data curation: ZS. Formal analysis: ZS, SV, JT, EG; Funding acquisition: RM, SV; Investigation: ZS, SV, JT, EG, PM, SM, JW, AG, PL, ND, RM; Methodology: ZS, SV, JT, EG, PM, SM, JW, AG, PL, CZ, ND, RM; Resources; Software; Supervision: ND, SV, RM; Writing – original draft: ZS, ND, SV, RM; Writing – review & editing: all authors.

## Funding

This work was supported by the 10.13039/100014653NIHR Manchester Biomedical Research Centre, 10.13039/501100000289CRUK (A27412 and A22902), the 10.13039/100005976Harry J Lloyd Charitable Trust (Career Development Award for SV) and the 10.13039/100010269Wellcome Trust (100282/Z/12/Z).

## Conflict of interest statement

RM is an expert witness for Pfizer. RM may benefit from drug discovery programmes that are commercialized. PL serves as a paid advisor/speaker for Bristol-Myers Squibb, Merck Sharp and Dohme, Roche, Novartis, Amgen, Pierre Fabre, Nektar, Melagenix. PL reports travel support from Bristol-Myers Squibb and Merck Sharp and Dohme, and receives research support from Bristol-Myers Squibb. AG received honoraria and consultancy fees from BMS and Novartis. All remaining authors have declared no conflicts of interest.
