# Supplementary figures and images for: T cell immune awakening in response to immunotherapy is age-dependent
Source: Eur J Cancer. 2022 Feb;162:11–21. doi: 10.1016/j.ejca.2021.11.015 (PMC8829752; doi:10.1016/j.ejca.2021.11.015)

A

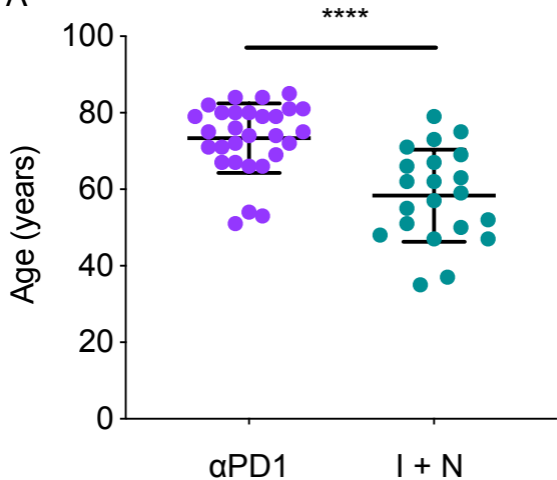

Supplement: Multimedia component 1 [file mmc1.pdf]

A

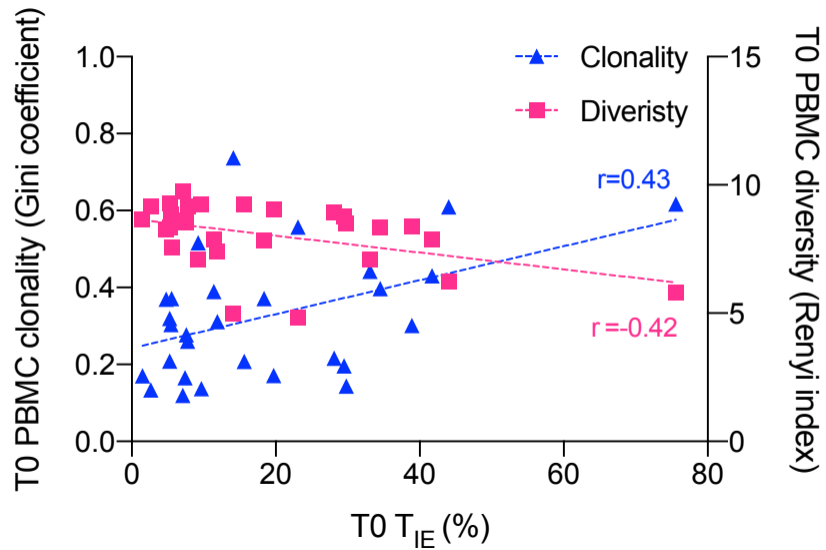

B

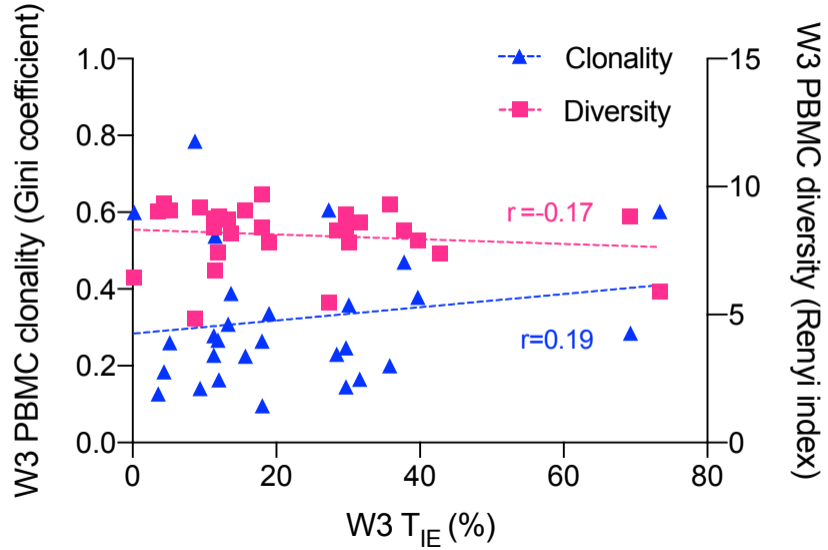

Supplement: Multimedia component 2 [file mmc2.pdf]
